# Association of white blood cell count to mean platelet volume ratio with type 2 diabetic peripheral neuropathy in a Chinese population: a cross-sectional study

**DOI:** 10.1186/s12902-024-01644-y

**Published:** 2024-07-29

**Authors:** Yu Wang, Ying Miao, Qin Wan

**Affiliations:** 1 Department of Cardiology, Luzhou People’s Hospital, Luzhou, China; 2 Department of Endocrinology and Metabolism, Affiliated Hospital of Southwest Medical University, Luzhou, China; 3 Metabolic Vascular Disease Key Laboratory of Sichuan Province, Luzhou, China; 4 Sichuan Clinical Research Center for Diabetes and Metabolism, Luzhou, China; 5 Sichuan Clinical Research Center for Nephropathy, Luzhou, China; 6 Cardiovascular and Metabolic Diseases Key Laboratory of Luzhou, Luzhou, China; 7 Southwest Medical University, Luzhou, China

**Keywords:** White blood cell count to mean platelet volume ratio, Diabetic peripheral neuropathy, Type 2 diabetes mellitus, Inflammation, National metabolic management center

## Abstract

**Background:**

The white blood cell count to mean platelet volume ratio (WMR) is considered a promising inflammatory marker, and its recognition is increasing. Inflammation is closely related to metabolic diseases such as diabetes and its complications. However, there are currently no reports on the correlation between WMR and type 2 diabetic peripheral neuropathy (DPN). This study aims to explore the correlation between WMR and DPN in type 2 diabetes patients. By understanding this association, we hope to provide a theoretical basis for preventing DPN through the improvement of inflammatory responses.

**Methods:**

This was a cross-sectional study involving 2515 patients with T2DM. Logistic regression analysis was conducted to assess the associations between WMR and DPN. Finally, the receiver operating characteristic curve (ROC curve) was employed to evaluate the predictive efficacy of WMR for DPN.

**Results:**

Patients in higher WMR quartiles exhibited increased presence of DPN. Additionally, WMR remained significantly associated with a higher odds ratio (OR) of DPN (OR 4.777, 95% confidence interval [CI] 1.296–17.610, *P* < 0.05) after multivariate adjustment. Moreover, receiver operating characteristic curve analysis indicated that the optimal cutoff value for WMR in predicting DPN presence was 0.5395 (sensitivity: 65.40%; specificity: 41.80%; and area under the curve [AUC]: 0.540).

**Conclusions:**

In patients with T2DM, WMR was significantly increased in DPN and independently associated with an increased risk of DPN presence in Chinese patients. This suggests that WMR may serve as a useful and reliable biomarker of DPN, highlighting the importance of paying more attention to T2DM patients with high WMR to further prevent and reduce the development of DPN and related unfavorable health outcomes.

## Introduction

Diabetic peripheral neuropathy (DPN) is one of the significant complications of diabetes, occurring in approximately 50% of diabetes patients [1]. DPN affects different parts of the nervous system and presents multiple manifestations. DPN affects different parts of the nervous system and presents with multiple manifestations. Currently, DPN lacks efficient treatment, resulting in various syndromes without a universally accepted classification [2]. These neuropathies are generally subdivided into focal/multifocal neuropathies, including diabetic amyotrophy, and symmetric polyneuropathies, such as sensorimotor polyneuropathy (DSPN). DSPN is the most common type, affecting about 30% of diabetic patients in hospital care and 25% of those in the community [3]. Therefore, early identification of high-risk individuals for DPN and early prevention are essential. Finding simple clinical indicators to assess the risk of DPN is crucial for the management of diabetic patients at the primary care level.

The pathogenesis of diabetic neuropathy is very complex. Hyperglycemia, dyslipidemia, and insulin resistance trigger a cascade of responses, activating pathways such as the polyol pathway, glycolysis pathway, hexosamine pathway, and advanced glycation end-product pathway. These activations enhance oxidative stress and inflammatory signals, leading to endoplasmic reticulum stress, mitochondrial dysfunction, DNA damage, and elevated inflammatory factor levels. Ultimately, this complex process substantiates the onset of diabetic neuropathy [1]. Chronic low-grade inflammation, considered a crucial link in diabetes complications, including DPN, has been shown to include low-grade intraneural inflammation as a facet of diabetic neuropathy [4]. It can also be understood as a type of inflammatory neuropathy, where blood-derived white blood cells actively participate in axonal degeneration, demyelination, or both, leading to sensory deficits [5]. The systemic inflammatory response, in which white blood cells (WBC) play a vital role, has been proven to be involved in the pathogenesis of diabetic microvascular complications [6]. Additionally, several studies have suggested that WBC count on admission is correlated with a greater degree of neurological impairment and unfavorable long-term outcomes [7]. Mean platelet volume (MPV), which reflects platelet size, can provide information on platelet function and activation [8]. In inflammatory conditions, MPV is also associated with an increased percentage of large platelets [9]. A study from Egypt showed that MPV was significantly higher among subjects with retinopathy, nephropathy, and neuropathy than in other subjects with diabetes who did not develop complications (*P* < 0.001). MPV may be considered a possible biomarker for the early detection of diabetic microvascular complications [6]. WMR, as a composite marker comprised of WBC count and MPV, is a novel inflammatory index [10]. Numerous studies have explored the correlation between WMR and cardiovascular diseases [11]. As far as we are aware, the relationship between WMR and DPN has never been determined, and the underlying mechanisms are less well understood.

Therefore, this cross-sectional study was conducted to investigate the relationship between WMR and the risk of the presence of DPN in Chinese adults with T2DM.

## Methods

### Study participants

**Fig. 1:**
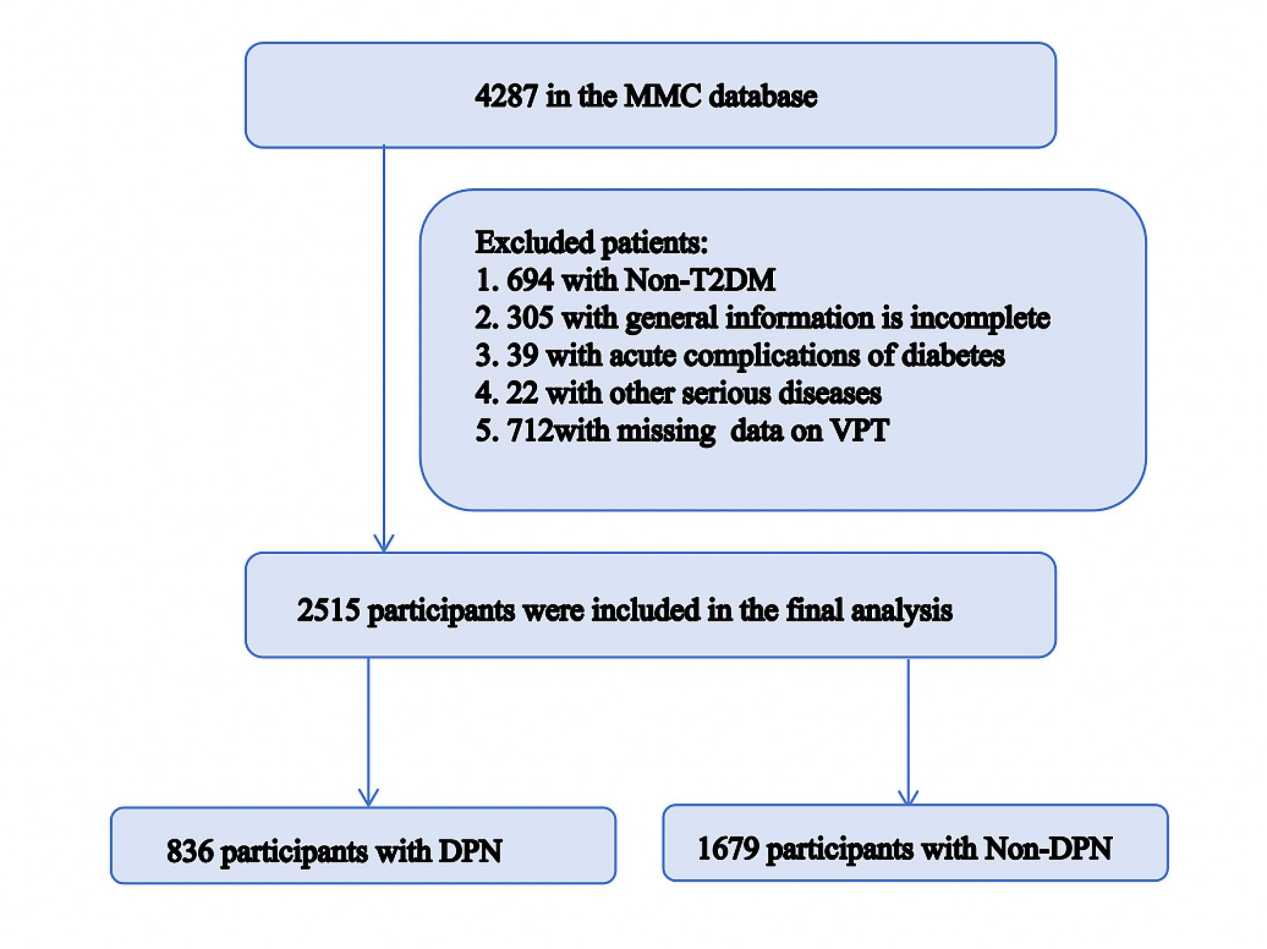
Flowchart for the selection of the analyzed study sample from the MMC database

Figure 1 illustrates the process of selecting study subjects from the National Metabolic Management Center (MMC) database. Following the application of inclusion and exclusion criteria, a total of 2515 T2DM patients, aged between 18 and 80 years, were identified from the Endocrinology Department at the Affiliated Hospital of Southwest Medical University. This selection occurred between September 2017 and January 2024. All participants underwent a comprehensive assessment, including a standardized questionnaire, anthropometric examination, physical examination, laboratory tests, and evaluation of diabetes-related complications. All patients were confirmed to be diagnosed with T2DM on the basis of the American Diabetes Association criteria with fasting plasma glucose (FPG) ≥ 7.0 mmol/l, or hemoglobin A1c (HbA1c) ≥ 6.5% or oral glucose tolerance test (OGTT) 2 h post-load plasma glucose ≥ 11.1 mmol/l or self-reported medical history [12]. Additionally, DPN was defined as vibration perception threshold (VPT) T ≥ 25 V and/or inability to feel the monofilament [13].

The inclusion criteria for participants were as follows: (1) Age above 18 years; (2) Diagnosed with T2DM according to the American Diabetes Association’s “Standards of Medical Care in Diabetes” (2020 version) [12]; (3) Completion of VPT examination. Conversely, the exclusion criteria encompassed: (1) Type 1 diabetes, gestational diabetes, or other specific types of diabetes (as the primary focus of this study is T2DM, other types were excluded); (2) Acute complications of diabetes, including diabetic ketoacidosis, hyperglycemic hyperosmolar state, hyperosmolar coma, and hypoglycemia (acute complications can significantly alter inflammatory markers, thus affecting study results); (3) Concurrent severe diseases such as tumors, liver failure, renal failure, etc. (these conditions or their treatments could affect blood test results, leading to potential confounding); (4) Missing or incomplete demographic or clinical characteristic data (lack of this data could introduce bias in multifactorial analyses).

### Data collection

Researchers with professional training collected participants’ demographic information, lifestyle details, medical history, and medication use data. The questionnaire used in this study was from MMC [14]. Physical examinations were conducted before breakfast, with participants dressed in light clothing and without shoes. Body weight in light clothes and without shoes was measured with a digital scale to the nearest 0.1 kg, and height was measured without shoes to the nearest 0.1 cm. Body mass index (BMI) was calculated as weight (kg) divided by height (m) squared. Circumferences of the head, neck, waist, and hips were measured. The waist-to-hip ratio (WHR) was calculated as the waist circumference divided by the hip circumference, and the waist-to-height ratio (WtHR) was calculated as the waist circumference divided by height. The systolic (SBP) and diastolic (DBP) blood pressure of the right arm were measured using an Omron blood pressure monitor. Three readings were taken and averaged.

Morning venous blood samples were collected from each participant after an overnight fast of at least 8 h. These samples were used to measure fasting plasma glucose (FPG), 2-hour postprandial glucose (2hPG), alanine aminotransferase (ALT), aspartate aminotransferase (AST), low-density lipoprotein cholesterol (LDL-C), high-density lipoprotein cholesterol (HDL-C), total cholesterol (TC), triglycerides (TG), uric acid (UA) were measured using a 7060 full-automatic biochemical analyzer (Hitachi) at the registered central laboratory located at the Affiliated Hospital of Southwestern Medical University. Glycated hemoglobin A1C (HbA1c) was measured by the anion exchange high performance liquid chromatography (Arkray Eluent 80 A). White blood cells (WBC), red blood cells (RBC), and mean platelet volume (MPV) were determined using an automated blood cell counter (Mindray BC-6800). WMR was calculated as WBC (*10^9^/L)/MPV (fL).

### VPT measurements

All patients with T2DM were asked if they experienced numbness, pain (prickling, stabbing, shooting, burning, or aching pain), and paresthesia (abnormal cold or heat sensation, allodynia, and hyperalgesia) in the toes, feet, legs, or upper limbs. An experienced physician then performed a neurological examination, including assessments of vibration, light touch, and Achilles tendon reflexes on both sides in the knee-standing position, noting their presence, weakening, or loss.

The vibration perception threshold (VPT) was assessed at the metatarsophalangeal joint of the big toe using a neurothesiometer (Bio-Thesiometer; Bio-Medical Instrument Co., Newbury, OH, USA). Initially, patients were instructed on how to recognize the vibration sensation by gradually increasing the amplitude from zero to maximum. The test then started from zero again, and patients were asked to indicate when they first felt the vibration. Measurements were taken on the plantar aspect of both big toes, three times consecutively for each toe, with the median of the three readings accepted as the VPT value.

Sensitivity to touch was tested using a 5.07/10-g Semmes-Weinstein monofilament (SWM) at four points on each foot: three on the plantar side and one on the dorsal side. The 10-g SWM was placed perpendicular to the skin, and pressure was applied until the filament just buckled, maintaining contact for 2 s. Inability to perceive the sensation at any site was considered abnormal. DPN was defined as VPT ≥ 25 V and/or inability to feel the monofilament, and participants were then divided into DPN and no DPN groups [15].

### Statistical analysis

Patients who could not be fully included in the study due to missing information were excluded. All patients with T2DM were divided into four groups based on WMR quartiles. Participant characteristics were presented as either mean (standard deviation) or median (interquartile range), depending on the distribution of continuous variables, while categorical variables were expressed as count (proportion). Continuous variable comparisons were conducted using Student’s t-test, Mann-Whitney U test, Kruskal-Wallis H test, or one-way ANOVA, depending on the normality of the data. Chi-square tests were employed for between-group comparisons of categorical variables. The correlation between WMR and DPN was analyzed using logistic regression models, presenting results as odds ratios (OR) with corresponding 95% confidence intervals (CI). All statistical analyses considered two-tailed *p*-values, with significance set at *p* < 0.05. The statistical software SPSS (version 26.0) was used for all analyses, and Forest plots were generated using GraphPad Prism (version 9.0.0).

## Results

### Association of WMR with clinical and laboratory characteristics in T2DM patients

The clinical and laboratory characteristics of 2,515 patients with T2DM (1,367 males, 54.40%, and 1,148 females, 45.60%) according to WMR quartiles are summarized in Table 1. Overall, the mean age was 56.70 ± 10.25 years, the mean BMI was 24.50 kg/m², and the mean WMR was 0.58. Patients in higher WMR quartiles tended to have higher levels of DBP, WHR, WtHR, WBC, RBC, PLT, WMR, serum Cr, UA, LDL-C, and urinary ACR, but lower levels of MPV and HDL-C compared to those in lower quartiles (*P* < 0.05). As WMR quartiles increased, the prevalence of DPN also increased, suggesting that higher WMR levels are associated with a higher risk of developing DPN, and this difference was statistically significant (*P* = 0.019).

**Table 1:** Association of WMR with clinical and laboratory characteristics in patients with T2DM

Variable	Total	Q1(*n* = 629) [0.0198–0.4764]	Q2(*n* = 628) (0.4764–0.5830]	Q3(*n* = 629) (0.5830–0.7203]	Q4(*n* = 629) (0.7203–1.9812]	*P*
Male(%)	1367(54.40%)	321(51.00%)	355(56.50%)	357(56.80%)	334(53.10%)	0.119
Age(years)	56.70 ± 10.25	57.12 ± 9.40	56.95 ± 10.14	56.26 ± 10.72	56.46 ± 10.69	0.404
Head circumference(cm)	56.00(55.00,57.50)	56.00(55.00,57.00)	56.00(55.00,58.00)	56.00(55.00,58.00)	56.00(55.00,57.00)	0.324
Neck circumference(cm)	37.00(34.00,39.00)	36.00(34.00,39.00)	37.00(34.00,39.00)	37.00(35.00,40.00)	37.00(34.00,39.75)	0.002
BMI(kg/m2)	24.50(22.40,26.80)	24.20(22.10,26.30)	24.60(22.53,26.78)	24.80(22.80,27.15)	24.40(22.20,26.90)	0.003
WHR	0.95(0.90,0.99)	0.93(0.88,0.98)	0.95(0.90,0.99)	0.95(0.91,0.99)	0.95(0.90,1.00)	< 0.001
WtHR	0.54(0.50,0.58)	0.53(0.49,0.56)	0.53(0.50,0.57)	0.54(0.51,0.58)	0.54(0.50,0.58)	< 0.001
SBP (mmHg)	132.50(120.00,148.00)	132.00(119.00,146.00)	131.00(120.00,144.00)	135.00(120.00,149.00)	133.00(120.00,152.00)	0.012
DBP(mmHg)	79.00(71.00,86.00)	78.00(71.00,85.00)	78.00(70.00,85.00)	79.00(72.00,87.00)	80.00(72.00,87.00)	0.010
FPG(mmol/L)	9.00(7.20,11.70)	9.00(7.20,11.70)	8.90(7.20,11.65)	8.80(7.10,11.40)	9.40(7.20,12.10)	0.152
2hPG(mmol/L)	13.80(11.10,17.70)	13.60(11.00,17.60)	13.80(11.20,17.70)	13.62(10.90,17.50)	14.20(11.60,17.83)	0.271
HOMA-IR	3.14(1.80,5.50)	2.62(1.48,4.93)	3.20(1.81,4.93)	3.19(1.89,4.90)	3.79(1.99,6.97)	0.002
HbA1c (%)	9.40(7.60,11.40)	9.50(7.50,11.50)	9.30(7.53,11.08)	9.30(7.58,11.20)	9.70(7.88,11.70)	0.010
WBC	6.50(5.39,7.81)	4.87(4.30,5.38)	5.98(5.46,6.57)	6.92(6.41,7.62)	8.99(7.92,10.16)	< 0.001
RBC	4.57(4.20,4.96)	4.48(4.11,4.86)	4.57(4.24,4.99)	4.65(4.21,5.03)	4.62(4.23,4.99)	< 0.001
HB	137.00(126.00,149.00)	135.00(124.00,147.00)	138.00(127.00,149.75)	139.20(126.00,152.00)	137.00(126.00,149.00)	0.001
PLT	204.00(165.00,246.00)	166.00(135.00,200.00)	193.00(161.00,228.75)	213.00(181.00,248.00)	247.00(209.00,291.50)	< 0.001
MPV	11.10(10.10,12.20)	12.10(11.10,13.10)	11.30(10.40,12.30)	10.80(10.00,11.70)	10.30(9.40,11.10)	< 0.001
WMR	0.58(0.48,0.72)	0.41(0.36,0.45)	0.53(0.50,0.56)	0.64(0.61,0.68)	0.85(0.77,0.97)	< 0.001
ALT	21.20(15.80,32.00)	21.70(15.90,32.50)	22.15(16.33,32.48)	21.20(15.60,32.75)	20.10(15.10,29.85)	0.005
AST	19.60(15.60,25.70)	20.00(16.35,26.75)	20.10(16.30,25.78)	19.80(15.60,25.85)	18.10(14.30,24.40)	< 0.001
GGT	26.00(17.30,43.50)	23.80(15.70,39.70)	26.05(17.35,45.40)	25.65(17.30,43.23)	28.50(19.08,46.83)	< 0.001
BUN	5.92(4.88,7.35)	5.76(4.82,6.89)	5.78(4.75,7.32)	6.07(4.97,7.39)	6.03(4.93,7.76)	0.001
Cr	61.90(50.90,77.40)	58.35(48.68,71.93)	60.50(50.18,75.28)	63.20(52.53,80.18)	65.25(53.33,81.35)	< 0.001
UA (µmol/L)	325.45(267.28,402.23)	307.60(258.93,380.03)	317.60(261.20,396.80)	327.45(270.98,402.00)	347.90(280.60,433.50)	< 0.001
TG(mmol/l)	1.77(1.21,2.78)	1.62(1.14,2.57)	1.80(1.21,2.88)	1.77(1.25,2.73)	1.88(1.28,2.86)	0.022
TC(mmol/l)	4.67(3.95,5.50)	4.67(3.96,5.46)	4.69(3.95,5.59)	4.65(3.95,5.47)	4.67(3.91,5.48)	0.704
HDL-C(mmol/l)	1.11(0.92,1.34)	1.14(0.94,1.39)	1.12(0.93,1.36)	1.10(0.91,1.32)	1.07(0.91,1.31)	0.005
LDL-C(mmol/l)	2.80(2.12,3.53)	2.71(2.06,3.44)	2.78(2.10,3.51)	2.78(2.15,3.52)	2.93(2.16,3.69)	0.031
Urinary ACR (mg/g)	18.70(9.00,62.53)	16.20(8.20,43.40)	16.45(7.90,43.85)	19.40(9.90,77.68)	25.70(11.15,108.40)	< 0.001
Smoking(%)						0.021
Never	1535(61.10%)	421(66.90%)	377(60.00%)	371(59.00%)	366(58.30%)	
Ever	539(21.40%)	105(16.70%)	142(22.60%)	142(22.60%)	150(23.90%)	
Current	440(17.50%)	103(16.40%)	109(17.40%)	116(18.40%)	112(17.80%)	
drinking(%)						0.274
Never	1378(54.90%)	347(55.20%)	342(54.50%)	336(53.50%)	353(56.20%)	
Ever	228(9.10%)	69(11.00%)	51(8.10%)	49(7.80%)	59(9.40%)	
Current	906(36.10%)	213(33.90%)	234(37.30%)	243(38.70%)	216(34.40%)	
DPN(%)	836(33.20%)	185(29.40%)	197(31.40%)	223(35.50%)	231(36.70%)	0.019

BMI: body mass index; WHR: waist-to-hip ratio; WtHR: waist-to-height ratio; SBP: systolic blood pressure; DBP: diastolic blood pressure; FPG: fasting plasma glucose; 2hPG: postprandial 2-hour plasma glucose; HOMA-IR: Homeostasis Model Assessment of Insulin Resistance; HbA1c: glycated hemoglobin A1c ; WBC: white blood cell; RBC: red blood cell; HB: hemoglobin: PLT: platelet; MPV: mean platelet volume; WMR: white blood cell count to mean platelet volume ratio; ALT: alanine aminotransferase; AST: aspartate aminotransferase; GGT: gamma-glutamyl transferase; BUN: Blood urea nitrogen; Cr: creatinine; UA: uric acid; TG: triglycerides; TC: total cholesterol; HDL-C: high-density lipoprotein cholesterol ; LDL-C: low-density lipoprotein cholesterol; ACR: albumin- to-creatinine ratio; DPN: diabetic peripheral neuropathy. ‘Q1’ for the first quartile: ‘Q2’ for the second quartile: ‘Q3’ for the third quartile: and ‘Q4’ for the fourth quartile

### Univariate and multivariate analysis of determinants of DPN in T2DM patients

Table 2 displays the associations of WMR and other variables with the risk of DPN presence. Univariate analysis revealed that age, head circumference, neck circumference, BMI, WtHR, SBP, DBP, FPG, 2hPG, HbA1c, ALT, AST, WBC, RBC, HB, PLT, BUN, TC, urinary ACR, smoking, drinking, and WMR were significantly associated with DPN presence (*P* < 0.05). Based on the results of univariate analysis, variables with significant associations were included in a binary logistic regression analysis, with WMR analyzed as a continuous variable. The results showed that age, BMI, SBP, FPG, HbA1c, WBC, RBC, BUN, urinary ACR, smoking, drinking, and WMR were significantly and independently associated with DPN presence (*P* < 0.05). Notably, each standard deviation increase in WMR was associated with a significant 4.777-fold increase in the odds of DPN (95% CI, 1.296–17.610, *P* < 0.05).

**Table 2:** Univariate and multivariate analysis of determinants of DPN in patients with T2DM

Variable	Univariate analysis		Multivariate analysis		
	Test the statistical value	*P*	B	OR(95%CI)	*P*
Gender(female vs. male)	0.830	0.773			
Age(years)	17.642	< 0.001	-0.040	0.961(0.948,0.974)	< 0.001
Head circumference(cm)	-3.441	0.001	-0.030	0.970(0.911,1.033)	0.347
Neck circumference(cm)	-2.240	0.025	-0.009	0.991(0.944,1.040)	0.710
BMI(kg/m^2^)	-4.413	< 0.001	-0.106	0.900(0.843,0.960)	0.001
WHR	-0.080	0.936			
WtHR	-2.601	0.009	2.768	15.922(0.481,527.135)	0.121
SBP (mmHg)	-5.631	< 0.001	0.014	1.015(1.005,1.024)	0.003
DBP(mmHg)	-6.099	< 0.001	0.007	1.008(0.992,1.024)	0.353
FPG(mmol/L)	-7.252	< 0.001	0.054	1.056(1.015,1.098)	0.007
2hPG(mmol/L)	-6.148	< 0.001	0.013	1.013(0.986,1.041)	0.345
HOMA-IR	-0.138	0.890			
HbA1c (%)	-11.358	< 0.001	0.183	1.201(1.138,1.267)	< 0.001
WBC	-3.199	0.001	-0.134	0.874(0.780,0.980)	0.021
RBC	-5.972	< 0.001	-0.346	0.708(0.522,0.960)	0.026
HB	-7.200	< 0.001	-0.01	0.990(0.979,1.001)	0.063
PLT	-4.090	< 0.001	0.002	1.002(1.000,1.004)	0.082
MPV	-0.749	0.454			
ALT	-6.415	< 0.001	0.002	1.002(0.998,1.006)	0.374
AST	-6.505	< 0.001	-0.004	0.996(0.989,1.004)	0.368
GGT	-1.966	0.049	0	1.000(0.999,1.001)	0.801
BUN	-5.117	< 0.001	0.07	1.072(1.020,1.127)	0.006
Cr	-1.321	0.186			
UA (µmol/L)	-1.324	0.185			
TG(mmol/l)	-0.476	0.634			
TC(mmol/l)	-2.353	0.019	-0.004	0.996(0.946,1.048)	0.867
HDL-C(mmol/l)	-0.996	0.319			
LDL-C(mmol/l)	-1.749	0.08			
Urinary ACR (mg/g)	-10.161	< 0.001	0	1.000(1.000,1.001)	< 0.001
Smoking	10.096	0.006			< 0.001
Never				Reference	
Ever			0.656	1.926(1.384,2.680)	< 0.001
Current			0.459	1.583(1.061,2.361)	0.024
drinking	8.741	0.013			0.176
Never				Reference	
Ever			0.047	1.049(0.679,1.619)	0.831
Current			-0.237	0.789(0.580,1.072)	0.129
WMR	-3.198	0.001	1.564	4.777(1.296,17.610)	0.019

### Association of WMR quartiles with the risk of presence of DPN in T2DM patients

**Fig. 2:**
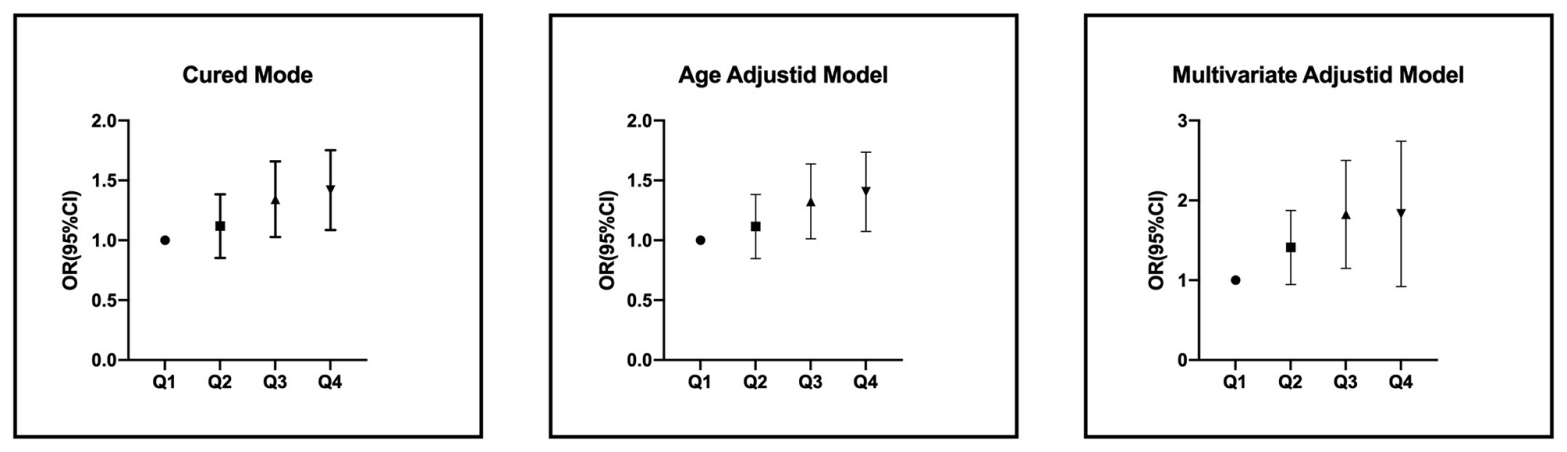
Forest plots for logistic regression analysis of WMR quartiles with the risk of presence of DPN in T2DM patients

As depicted in Table 3; Fig. 2, WMR was grouped as a categorical variable and analyzed using binary logistic regression. The results showed that the risk of diabetic peripheral neuropathy (DPN) increased progressively with higher WMR quartiles among the study subjects. Compared to the lower quartile (Q1), the higher quartiles of WMR (Q3 and Q4) were significantly associated with increased odds of DPN (OR = 1.318 and 1.393, respectively). After adjusting for age, the higher quartiles of WMR (Q3 and Q4) remained significantly associated with increased odds of DPN compared to the lower quartile (Q1) (OR = 1.300 and 1.379, respectively). Further adjustment for head circumference, neck circumference, BMI, WtHR, SBP, DBP, FPG, 2hPG, HbA1c, ALT, AST, WBC, RBC, HB, PLT, BUN, TC, urinary ACR, smoking, and drinking still showed that the higher quartiles of WMR (Q3 and Q4) were significantly associated with increased odds of DPN compared to the lower quartile (Q1) (OR = 1.742 and 1.681, respectively). These differences were statistically significant (*P* < 0.05). This suggests that when WMR is analyzed as a categorical variable, higher quartiles are associated with an increased risk of DPN compared to the Q1 group.

**Table 3:** Association of WMR quartiles with the risk of presence of DPN in patients with T2DM

WMR	Model1		Model2		Model3	
	OR(95%CI)	*P*	OR(95%CI)	*P*	OR(95%CI)	*P*
Q1	Reference		Reference		Reference	
Q2	1.097(0.863,1.395)	0.451	1.094(0.859,1.392)	0.467	1.359(0.974,1.897)	0.071
Q3	1.318(1.040,1.671)	0.022	1.300(1.025,1.649)	0.03	1.742(1.195,2.539)	0.004
Q4	1.393(1.100,1.764)	0.006	1.379(1.088,1.748)	0.008	1.681(1.007,2.806)	0.047

Model 1: Unadjusted;

Model 2: Adjusted for age;

Model 3: Adjusted for age, head circumference, neck circumference, BMI, WtHR, SBP, DBP, FPG, 2hPG, HbA1c, ALT, AST, WBC, RBC, HB, PLT, BUN, TC, urinary ACR, smoking and drinking

### Predictive value of WMR in screening for the presence of DPN in T2DM patients

**Fig. 3:**
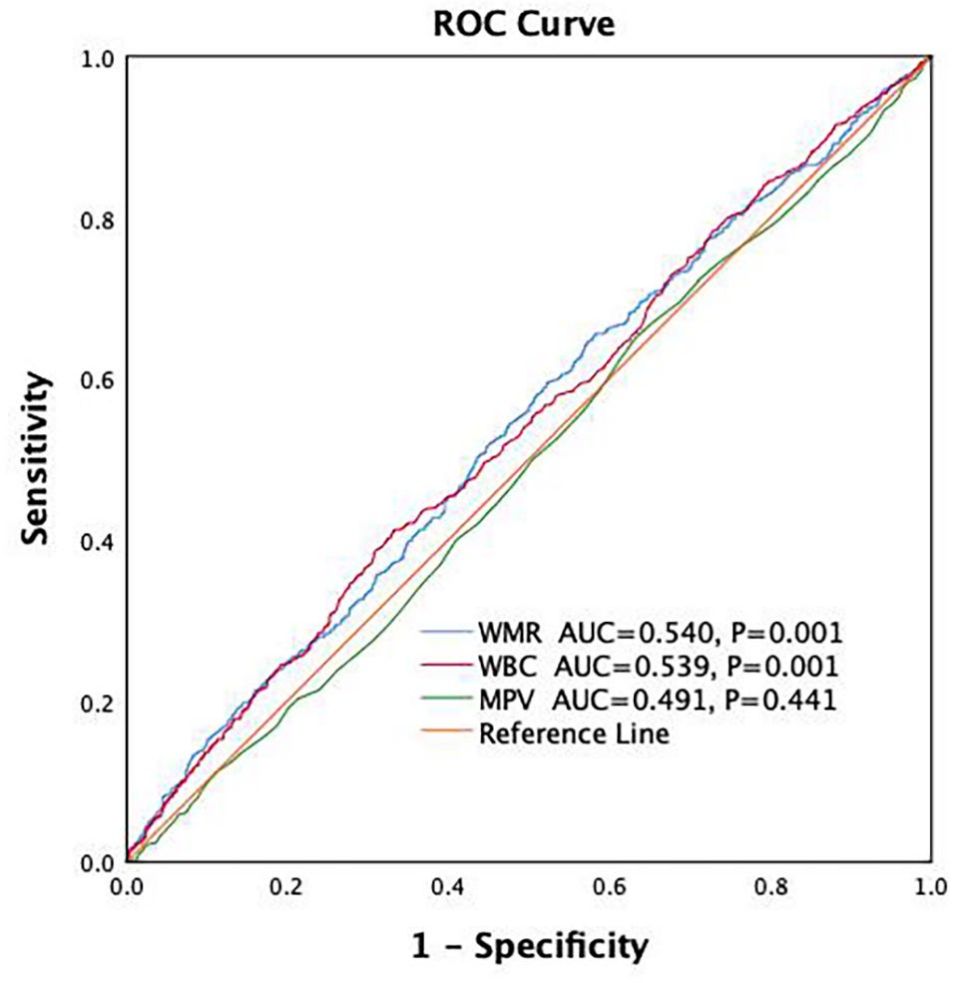
Receiver operating characteristics (ROC) curve analysis of WMR to inidicate DPN

To explore the predictive value of WMR for DPN, we analyzed the ROC curves. The analysis showed that WMR (AUC = 0.540, 95%CI = 0.516–0.564, *P* = 0.001) has a better predictive ability for DPN compared to WBC (AUC = 0.539, 95%CI = 0.515–0.563, *P* = 0.001) and MPV (AUC = 0.491, 95%CI = 0.467–0.514, *P* = 0.441). The optimal cutoff value for WMR to predict the presence of DPN was determined to be 0.5395, with a corresponding sensitivity of 65.40% and a specificity of 41.80% (Fig. 3). Further DeLong tests were conducted to evaluate the predictive values of WBC and WMR for DPN. The results showed *P* = 0.869, indicating that there is no statistically significant difference between WBC and WMR in predicting DPN based on the ROC analysis.

## Discussion

To our knowledge, this study represents the first investigation into the relationship between WMR and the risk of DPN presence. In this cross-sectional study involving 2515 adult patients with T2DM, we observed a positive correlation between WMR and the occurrence of DPN. Patients in higher WMR quartiles exhibited a greater presence of DPN, with WMR emerging as an independent determinant of DPN presence following multivariate adjustment. Moreover, the risk of DPN presence increased progressively with higher WMR quartiles. ROC curve analysis showed that the AUC of WMR in predicting DPN was higher than that of WBC and MPV. However, further DeLong test results indicated that there was no statistically significant difference between WMR and WBC in predicting DPN. Previous binary logistic regression analysis results demonstrated that, after adjusting for multiple factors, the risk of DPN decreased with increasing WBC (OR < 1). Considering both the logistic regression analysis and ROC curve analysis, we believe that the correlation between WBC and DPN is highly influenced by other confounding factors. In contrast, WMR appears to be more stable than WBC, making WMR a more suitable predictive indicator for DPN. Additionally, ROC curve analysis results suggest the optimal cutoff value for WMR to predict the presence of DPN was determined to be 0.5395. While the ROC curve may indicate low sensitivity and specificity, this suggests that the biomarker may not be useful for individual diagnosis but could have a role in population-level screening. These findings suggest that elevated WMR may correlate with DPN presence in hospitalized Chinese T2DM patients, potentially serving as an additional risk indicator for DPN.

As mentioned earlier, the impact of chronic low-grade inflammation on the progression of DPN has been widely recognized [16–18]. White blood cells (WBCs) typically act as the initial responders to acute inflammation and aid in its resolution [19]. However, in the context of chronic inflammation, the role of WBCs is less clear and can be either beneficial or detrimental, potentially causing tissue damage and amplifying the immune response [20–22]. Emerging evidence indicates that WBCs and the cytokines they release play significant roles in various chronic diseases, including atherosclerosis, diabetes mellitus, nonalcoholic fatty liver disease, and autoimmune disorders [23–31]. Moreover, in individuals with Type 1 diabetes mellitus (T1DM) who experience pain compared to those without, there is a proportional increase in CD4 + central memory T cells and an absolute increase in classical and nonclassical monocytes among peripheral blood immunophenotypes [32]. T-cell infiltration in the dorsal root ganglia is prominent in the late phase of the disease [33]. Additionally, sciatic nerve samples from Type 2 diabetes mellitus (T2DM) patients show macrophage and T-cell infiltration and increased expression of clusters of cells expressing CD40 [34].

Platelets play a role in the formation of atherosclerosis and atherothrombosis [35]. They not only participate in clot formation but are also involved in inflammatory processes [36]. During the inflammatory course, the proportion of larger platelets was found to increase, possibly due to the synthesis of factors promoting coagulation and inflammation, and a release of platelets stored in the spleen [37]. Simultaneously, these platelets are rapidly recruited to the site of inflammation, where they may become activated and worn down, potentially contributing to the decrease in MPV observed in patients with inflammatory conditions [38].

Inflammation is an adaptive response triggered by noxious stimuli and conditions. Tissue stress or malfunction may induce mild chronic inflammatory activity by activating the innate immune system. Evidence shows that long-term mild inflammation has a significant negative impact on the pathogenesis of diabetic neuropathy. Glucose, lipoproteins, oxidized and glycated proteins bind with various receptors on neurons, including transporters that internalize glucose and lipids, leading to their accumulation in cells and disruption of mitochondrial metabolic pathways, which also initiate inflammatory signals [1]. A novel inflammatory indicator, WMR (a composite marker comprised of WBC count and MPV), has been recognized in other literature as a relatively new marker for prognosis in atherosclerotic diseases and myocardial infarction [10]. Experimental and epidemiological studies have demonstrated the critical role of atherosclerotic vascular disease in the development and progression of DPN, suggesting an interaction between these biomarkers related to the inflammatory response [15]. Low-grade intraneural inflammation is a facet of diabetic neuropathy. These mild inflammatory processes likely represent a common terminal pathway associated with the degeneration of intraepidermal nerve fibers. Dysregulation of glucose and lipid metabolism, as well as obesity-induced mild inflammation, may contribute to peripheral neuropathy [4]. In our study, we observed an association between increased WMR and the risk of diabetic peripheral neuropathy (DPN), potentially explained by inflammatory mechanisms. Our findings are akin to those of O’Brien JA et al., who noted increased CD4 + central memory T cells, classical, and nonclassical monocytes in peripheral blood immunophenotypes of Type 1 diabetes mellitus patients with pain compared to those without [32], though our study focused on Type 2 diabetes mellitus patients. Consistent with research from Egypt, a decrease in mean platelet volume (MPV) was associated with a heightened risk of DPN [6]. Therefore, during inflammation, elevated white blood cell (WBC) counts and reduced MPV contribute to an increased WMR. For patients with subtle changes in WBC or MPV, WMR may offer superior clinical value, elucidating its predictive advantage over WBC or MPV alone.

Our findings suggest that the elevated WMR may be attributed to increased WBC count and decreased MPV during inflammation. Moreover, we identified a significant positive correlation between WMR and DPN. Additionally, WMR can be easily calculated using two inexpensive and readily available biomarkers obtained from a complete blood cell count test, which could assist clinicians in screening the high-risk population for DPN. It’s noteworthy that in subsequent studies, if the inflammatory pathogenesis can be further supported by data from human tissues and intervention studies, anti-inflammatory compounds with different mechanisms of action may emerge as potential candidates for the treatment or prevention of diabetic neuropathy [4].

However, it should be noted that several limitations still exist in our study. Firstly, this study is cross-sectional, and therefore, the causative link between WMR and DPN cannot be determined. Secondly, the potential mechanism of the association between WMR and DPN requires further prospective large-scale study. Despite these limitations, the relatively large sample size strengthens the validity of our results. Considering that WMR can be easily calculated from routine indicators, it is readily available for use in clinical practice, particularly in large screening procedures.

## Conclusions

In patients with T2DM, WMR was significantly increased in DPN and independently associated with an increased risk of DPN presence in Chinese patients. This suggests that WMR may serve as a useful and reliable biomarker of DPN, highlighting the importance of paying more attention to T2DM patients with high WMR to further prevent and reduce the development of DPN and related unfavorable health outcomes.

## Data Availability

The datasets used and/or analysed during the current study available from the corresponding author on reasonable request.

## References

[1] Cheng Y, Chen Y, Li K, et al. How inflammation dictates diabetic peripheral neuropathy: an enlightening review. CNS Neurosci Ther [J]. 2024;30:e14477. 37795833 10.1111/cns.14477 PMC11017439

[2] Selvarajah D, Kar D, Khunti K, et al. Diabetic peripheral neuropathy: advances in diagnosis and strategies for screening and early intervention. Lancet Diabetes Endocrinol [J]. 2019;7:938–48. 31624024 10.1016/S2213-8587(19)30081-6

[3] Sloan G, Selvarajah D, Tesfaye S. Pathogenesis, diagnosis and clinical management of diabetic sensorimotor peripheral neuropathy. Nat Rev Endocrinol [J]. 2021;17:400–20. 34050323 10.1038/s41574-021-00496-z

[4] Baum P, Toyka KV, Blüher M et al. 2021. Inflammatory mechanisms in the pathophysiology of Diabetic Peripheral Neuropathy (DN)-New aspects. Int J Mol Sci [J], 22. 10.3390/ijms221910835 PMC8509236 34639176

[5] Ubogu EE. Inflammatory neuropathies: pathology, molecular markers and targets for specific therapeutic intervention. Acta Neuropathol [J]. 2015;130:445–68. 26264608 10.1007/s00401-015-1466-4 PMC4575885

[6] Mi AE, Abdallah N, Eldars W. Mean platelet volume and platelet distribution Width correlate with microvascular complications in Egyptian people with type 2 diabetes Mellitus. Curr Diabetes Rev [J]. 2021;17:e080621193947. 34102979 10.2174/1573399817666210608121024

[7] DeLong JH, Ohashi SN, O’Connor KC, et al. Inflammatory responses after ischemic stroke. Semin Immunopathol [J]. 2022;44:625–48. 35767089 10.1007/s00281-022-00943-7

[8] Akın H, Bilge Ö, Yavuz B, et al. The relationship between mean platelet volume and resistant hypertension. Clin Exp Hypertens [J]. 2022;44:228–32. 34974786 10.1080/10641963.2021.2022686

[9] Xu B, Zhang Y, Chen G, et al. Association of mean platelet volume/lymphocyte ratio with inflammation in non-dialysis patients with chronic kidney disease stages 1–4: a retrospective study. Front Immunol [J]. 2022;13:1041356. 36466904 10.3389/fimmu.2022.1041356 PMC9716279

[10] Weng Y, Gao Y, Zhao M, et al. The white blood cell count to mean platelet volume ratio for ischemic stroke patients after intravenous thrombolysis. Front Immunol [J]. 2022;13:995911. 36263052 10.3389/fimmu.2022.995911 PMC9574706

[11] Staszewski J, Pogoda A, Data K, et al. The mean platelet volume on admission predicts unfavorable stroke outcomes in patients treated with IV thrombolysis. Clin Interv Aging [J]. 2019;14:493–503. 30880930 10.2147/CIA.S195451 PMC6398411

[12] 2020. 2. Classification and diagnosis of diabetes: standards of Medical Care in Diabetes-2020. Diabetes Care [J], 43: S14–31. 10.2337/dc20-S002 31862745

[13] Lu B, Yang Z, Wang M, et al. High prevalence of diabetic neuropathy in population-based patients diagnosed with type 2 diabetes in the Shanghai downtown. Diabetes Res Clin Pract [J]. 2010;88:289–94. 20359765 10.1016/j.diabres.2010.02.002

[14] Wu Y, Wan Q, Xu Y, et al. Lower visceral Fat Area in patients with type 2 Diabetic Peripheral Neuropathy. Diabetes Metab Syndr Obes [J]. 2022;15:3639–54. 36439295 10.2147/DMSO.S388330 PMC9694982

[15] Yan P, Wu Y, Dan X, et al. Aspartate aminotransferase/alanine aminotransferase ratio was associated with type 2 diabetic peripheral neuropathy in a Chinese population: a cross-sectional study. Front Endocrinol (Lausanne) [J]. 2023;14:1064125. 36909318 10.3389/fendo.2023.1064125 PMC9998996

[16] Cheng Y, Chen Y, Li K, et al. How inflammation dictates diabetic peripheral neuropathy: an enlightening review. CNS Neurosci Ther [J]; 2023. 10.1111/cns.14477 PMC11017439 37795833

[17] Yu FX, Lee PSY, Yang L, et al. The impact of sensory neuropathy and inflammation on epithelial wound healing in diabetic corneas. Prog Retin Eye Res [J]. 2022;89:101039. 34991965 10.1016/j.preteyeres.2021.101039 PMC9250553

[18] Yang J, Wei Y, Zhao T, et al. Magnolol effectively ameliorates diabetic peripheral neuropathy in mice. Phytomedicine [J]. 2022;107:154434. 36122436 10.1016/j.phymed.2022.154434

[19] Foy BH, Sundt TM, Carlson JCT, et al. Human acute inflammatory recovery is defined by co-regulatory dynamics of white blood cell and platelet populations. Nat Commun [J]. 2022;13:4705. 35995789 10.1038/s41467-022-32222-2 PMC9395541

[20] Barnes PJ. Inflammatory mechanisms in patients with chronic obstructive pulmonary disease. J Allergy Clin Immunol [J]. 2016;138:16–27. 27373322 10.1016/j.jaci.2016.05.011

[21] Zhang S, Gang X, Yang S, et al. The alterations in and the role of the Th17/Treg Balance in Metabolic diseases. Front Immunol [J]. 2021;12:678355. 34322117 10.3389/fimmu.2021.678355 PMC8311559

[22] England C. 2023. Physiology in Perspective. Physiology (Bethesda) [J], 38: 160. 10.1152/physiol.00014.2023 37307527

[23] Xiong J, Li Z, Tang H, et al. Bulk and single-cell characterisation of the immune heterogeneity of atherosclerosis identifies novel targets for immunotherapy. BMC Biol [J]. 2023;21:46. 36855107 10.1186/s12915-023-01540-2 PMC9974063

[24] Liu Y, Lai X, Guo W, et al. Total White Blood Cell Count Mediated the Association between Increased Arterial Stiffness and risk of type 2 diabetes Mellitus in Chinese adults. Arterioscler Thromb Vasc Biol [J]. 2020;40:1009–15. 32078369 10.1161/ATVBAHA.119.313880

[25] Hammerich L, Tacke F. Hepatic inflammatory responses in liver fibrosis. Nat Rev Gastroenterol Hepatol [J]. 2023;20:633–46. 37400694 10.1038/s41575-023-00807-x

[26] Herrero-Cervera A, Soehnlein O, Kenne E. Neutrophils in chronic inflammatory diseases. Cell Mol Immunol [J]. 2022;19:177–91. 35039631 10.1038/s41423-021-00832-3 PMC8803838

[27] Fazeli P, Kalani M, Hosseini M. T memory stem cell characteristics in autoimmune diseases and their promising therapeutic values. Front Immunol [J]. 2023;14:1204231. 37497231 10.3389/fimmu.2023.1204231 PMC10366905

[28] Cai X, Wang M, Liu S, et al. Establishment and validation of a nomogram that predicts the risk of type 2 diabetes in obese patients with non-alcoholic fatty liver disease: a longitudinal observational study. Am J Transl Res [J]. 2022;14:4505–14. 35958467 PMC9360847

[29] Cai X, Gao J, Liu S et al. 2022. Hepatic Steatosis Index and the Risk of Type 2 Diabetes Mellitus in China: Insights from a General Population-Based Cohort Study. Dis Markers [J], 2022: 3150380. 10.1155/2022/3150380 PMC9365599 35968500

[30] Cai X, Zhu Q, Cao Y et al. 2021. A Prediction Model Based on Noninvasive Indicators to Predict the 8-Year Incidence of Type 2 Diabetes in Patients with Nonalcoholic Fatty Liver Disease: A Population-Based Retrospective Cohort Study. Biomed Res Int [J], 2021: 5527460. 10.1155/2021/5527460 PMC8140840 34095297

[31] Cai XT, Ji LW, Liu SS, et al. Derivation and validation of a Prediction Model for Predicting the 5-Year incidence of type 2 diabetes in non-obese adults: a Population-based Cohort Study. Diabetes Metab Syndr Obes [J]. 2021;14:2087–101. 34007195 10.2147/DMSO.S304994 PMC8123981

[32] O’Brien JA, McGuire HM, Shinko D, et al. T lymphocyte and monocyte subsets are dysregulated in type 1 diabetes patients with peripheral neuropathic pain. Brain Behav Immun Health [J]. 2021;15:100283. 34589782 10.1016/j.bbih.2021.100283 PMC8474166

[33] Agarwal N, Helmstädter J, Rojas DR, et al. Evoked hypoalgesia is accompanied by tonic pain and immune cell infiltration in the dorsal root ganglia at late stages of diabetic neuropathy in mice. Mol Pain [J]. 2018;14:1744806918817975. 30453826 10.1177/1744806918817975 PMC6311571

[34] Kan HW, Hsieh JH, Chien HF et al. 2018. CD40-mediated HIF-1α expression underlying microangiopathy in diabetic nerve pathology. Dis Model Mech [J], 11. 10.1242/dmm.033647 PMC5963861 29549140

[35] Gawaz M, Geisler T, Borst O. Current concepts and novel targets for antiplatelet therapy. Nat Rev Cardiol [J]. 2023;20:583–99. 37016032 10.1038/s41569-023-00854-6

[36] Khodadi E. Platelet function in Cardiovascular Disease: activation of molecules and activation by molecules. Cardiovasc Toxicol [J]. 2020;20:1–10. 31784932 10.1007/s12012-019-09555-4

[37] Schwertz H, Köster S, Kahr WH, et al. Anucleate platelets generate progeny. Blood [J]. 2010;115:3801–9. 20086251 10.1182/blood-2009-08-239558 PMC2865870

[38] Kamath S, Blann AD, Lip GY. Platelet activation: assessment and quantification. Eur Heart J [J]. 2001;22:1561–71. 11492985 10.1053/euhj.2000.2515

